# Long-Chain Acyl-CoA Synthetase is Associated with the Growth of *Malassezia* spp.

**DOI:** 10.3390/jof5040088

**Published:** 2019-09-21

**Authors:** Tenagy, Kengo Tejima, Xinyue Chen, Shun Iwatani, Susumu Kajiwara

**Affiliations:** School of Life Science and Technology, Tokyo Institute of Technology, J3-7, 4259 Nagatsuta, Midori-ku, Yokohama, Kanagawa 226-8501, Japan; tenagy@yahoo.com (T.); jianwu.tjm@gmail.com (K.T.); chen.x.ac@m.titech.ac.jp (X.C.); siwatani@bio.titech.ac.jp (S.I.)

**Keywords:** *Malassezia*, acyl-CoA synthetase, fatty acid uptake

## Abstract

The lipophilic fungal pathogen *Malassezia* spp. must acquire long-chain fatty acids (LCFAs) from outside the cell. To clarify the mechanism of LCFA acquisition, we investigated fatty acid uptake by this fungus and identified the long-chain acyl-CoA synthetase (ACS) gene *FAA1* in three *Malassezia* spp.: *M*. *globosa*, *M. pachydermatis*, and *M. sympodialis.* These *FAA1* genes could compensate for the double mutation of *FAA1* and *FAA4* in *Saccharomyces cerevisiae*, suggesting that *Malassezia* Faa1 protein recognizes exogenous LCFAs. *Mg*Faa1p and *Mp*Faa1p utilized a medium-chain fatty acid, lauric acid (C12:0). Interestingly, the ACS inhibitor, triacsin C, affected the activity of the *Malassezia* Faa1 proteins but not that of *S. cerevisiae*. Triacsin C also reduced the growth of *M. globosa*, *M. pachydermatis*, and *M. sympodialis*. These results suggest that triacsin C and its derivatives are potential compounds for the development of new anti-*Malassezia* drugs.

## 1. Introduction

*Malassezia* spp. are basidiomycetous fungi that commonly inhabit the skin and scalp of humans and homothermic animals. Several *Malassezia* species can cause infectious diseases, such as atopic dermatitis, seborrheic dermatitis, and pityriasis versicolor in humans, particularly in immunocompromised individuals. By contrast, *M. pachydermatis* is found primarily in animals and causes otitis externa in dogs [1,2,3,4]. To date, 18 *Malassezia* species have been identified, all of which lack fatty acid synthetase (FAS) genes in their genome [5,6]. Because of their inability to synthesize fatty acids de novo, it is necessary for *Malassezia* species to obtain long-chain fatty acids (LCFAs) from outside the cell, such that most *Malassezia* spp. are lipid dependent. Although *M. pachydermatis* is capable of growth on rich media such as yeast peptone dextrose (YPD) and Sabouraud dextrose agar, and was previously thought to be lipid independent, it was recently reported that *M. pachydermatis* cannot grow on synthetic media lacking fatty acids. Because rich media contain a small amount of fatty acids, it is assumed that *M. pachydermatis* efficiently utilizes fatty acids [7].

To obtain exogenous fatty acids, *Malassezia* spp. produce and release many lipases [2,8,9,10]. Secretion of lipases to degrade exogenous lipids into fatty acids is a well-known pathogenic trait of *Malassezia* spp [9]. Although the acquired fatty acids are utilized for the growth and proliferation of the fungus, some of the unsaturated fatty acids can cause inflammation of the skin in animals and humans [9,11]. Thus, utilization of exogenous fatty acids is associated with both the pathogenicity and proliferation of *Malassezia* spp.

Long-chain acyl-CoA synthetases (ACSs) activate LCFAs into corresponding acyl-CoA esters via thioesterification of the fatty acids with coenzyme A. In the budding yeast *Saccharomyces cerevisiae*, six ACSs (Faa1p, Faa2p, Faa3p, Faa4p, Fat1p, and Fat2p) have been characterized, and both Faa1p and Faa4p are known to activate LCFAs [12,13]. Faa1p and Faa4p are paralogs that primarily recognize fatty acids containing 14–16 carbons [12]. Faa1p localizes in the endoplasmic reticulum, plasma membrane, lipid particles, and mitochondria, whereas Faa4p localizes in the cytosol and lipid particles. Another ACS, Faa2p, is involved in the import of short- and medium-chain fatty acids into the peroxisome in *S. cerevisiae.* Faa2p and Fat1p reportedly function with the half-size ABC transporter proteins Pxa1p-Pxa1p in transporting very-long-chain fatty acids into the yeast peroxisome [14,15]. The observation that *faa1* and *faa4* double-null mutants of *S. cerevisiae* cannot grow on medium supplemented with cerulenin (a FAS inhibitor) and LCFAs indicates that Faa1p and Faa4p are essential for the growth of this yeast. By contrast, *FAA2*, *FAT1*, and *FAT2* null mutants are capable of growing on medium containing cerulenin and LCFAs [16].

For the clinical treatment of infectious diseases caused by *Malassezia* spp., azole drugs such as itraconazole, fluconazole, and ketoconazole, which interfere with sterol synthesis, are frequently used. However, a number of strains of other pathogenic fungi that are resistant to azole drugs have been isolated, like azole-resistant *Candida parapsilosis* and *C. albicans* [17,18], and azole-resistant *Cryptococcus neoformans* [19]. Various azole-resistant *M. pachydermatis* strains have also been isolated recently [20,21]. Therefore, as new drugs that inhibit other critical fungal metabolic pathways are needed, drugs that inhibit the activity of Faa proteins are potential alternatives. Triacsin C inhibits bacteria, mammals, and protozoan ACSs [22,23,24,25,26,27]. A previous report also indicated that triacsin C inhibits the activity of Faa4p (IC_50_ = 4.5 ± 0.5 μM), but not of Faa1p (IC_50_ > 500 μM) in *S. cerevisiae* [28].

Here, we identified homologs of the *S. cerevisiae FAA1* and *FAA4* genes in *M. globosa*, *M. pachydermatis*, and *M. sympodialis*. Introduction of these genes into an *S. cerevisiae faa1faa4* mutant (SCFAA1-4) restored its growth in a medium containing cerulenin and LCFAs. The result suggested that the *Malassezia* genes *MpFAA1*, *MgFAA1*, and *MsFAA1* encode an ACS in *S. cerevisiae.* The expression of the *MpFAA1* gene in *M. pachydermatis* was induced in a medium containing C16:0 and C18:1 fatty acids. Interestingly, triacsin C inhibited the growth of *S. cerevisiae faa1faa4* mutant harboring *Malassezia FAA1* genes, but not the growth of inherent *S. cerevisiae*.

## 2. Materials and Methods

### 2.1. Yeast Strains, Media, and Growth Conditions

Three *Malassezia* species were used in this study: *M. pachydermatis* CBS1879^NT^ (purchased from Centraalbureau voor Schimmelcultures, Utrecht, The Netherlands), *M. globosa* CBS7966^T^, and *M. sympodialis* CBS7222^T^ (both kindly provided by Prof. Somay Yamagata Murayama, Kitasato University, Japan). The yeasts were revived and subcultured at 32 °C on MLNA (modified Leeming-Notman agar—1% Bacto^TM^ peptone (BD Difco), 1% glucose, 0.2% yeast extract, 0.8% desiccated ox bile, 1% glycerol, 0.05% glycerol monostearate, 0.5% Tween 60, 1.5% agar, and 2% olive oil) every 2–4 weeks for maintenance. *Malassezia* were typically cultured in a liquid medium using mDixon (3.6% malt extract (Kyokuto, Co. Ltd.), 0.6% Bacto^TM^ peptone (BD Difco), 2% desiccated ox bile, 0.1% Tween 40, 0.2% glycerol, and 0.2% oleic acid) at 32 °C.

Strains of *S. cerevisiae* used in this study are listed in Table S1. *S. cerevisiae* strains IFO10150 and *faa1faa4* mutant (SCFAA1-4) were cultured in YPD medium (2% glucose, 2% peptone, and 1% yeast extract) and grown at 30 °C in CM ura^−^ medium (0.67% yeast nitrogen base without amino acids (Difco), 0.12% uracil drop-out [29]) containing different fatty acids (C10:0, C12:0, C14:0, C16:0, C18:0, and C18:1) at 0.5 mM and 5 μg/mL cerulenin (Wako, Japan). Most chemicals used for media preparation were purchased from Nacalai Tesque Inc. (Kyoto, Japan).

### 2.2. Plasmids

Plasmids used in this study are listed in Table S2, and the primers used to construct the plasmids are listed in Table S3. General DNA manipulations were performed according to the protocols reported by Sambrook et al. (1989) [30].

Plasmid pTRS7 harboring the *ADH1* promoter and terminator was used as an expression vector to insert the *Malassezia FAA1* genes, *ScFAA1* and *ScFAA4*, into SCFAA1-4. *MpFAA1* was amplified from *M. pachydermatis* 1879^NT^ genomic DNA extracted by PCR using primers MpFAA1-F and MpFAA1-R and then digested with *Sal*I and *Sma*I. *MgFAA1* was amplified from *M. globosa* 7966^T^ genomic DNA by PCR using primers MgFAA1-F and MgFAA1-R and then cut with *Eco*RI and *Bam*HI. *MsFAA1* was amplified from *M. sympodialis* 7222^T^ genomic DNA using primers MsFAA1-F and MsFAA1-R and then cut using *Sma*I and *Spe*I. For the control in this paper, a revertant strain containing one of the original *S. cerevisiae FAA1* and *FAA4* genes was constructed. *ScFAA1* was amplified from *S. cerevisiae* IFO10150 genomic DNA using primers ScFAA1-F and ScFAA1-R, and then cut by *Sal*I and *Not*I. On the other hand, *ScFAA4* was amplified from the same *S. cerevisiae* strain using primers ScFAA4-F and ScFAA4-R, and then cut by *BamH*I and *Not*I. The amplified DNA fragments were cloned into digested sites in pTRS7, and the resulting plasmids were designated pTRS7-MpFAA1, pTRS7-MgFAA1, pTRS7-MsFAA1, pTRS7-ScFAA1, and pTRS7-ScFAA4, respectively. These plasmids were cloned into *Escherichia coli* DH5α (Agilent Technologies, Santa Clara, CA, USA), extracted, and transformed into the SCFAA1-4 strain.

### 2.3. Transformation of S. cerevisiae

*S. cerevisiae* was transformed using a previously described method [31]. *URA3* was used as a marker to select transformants harboring the constructed plasmids encoding *Malassezia* genes. The successful transformants were confirmed by colony PCR using the same primers used for gene amplification.

### 2.4. Triacsin C Minimum Inhibitory Concentration (MIC) Analysis

Cells of mDixon-cultured *Malassezia* were collected, washed, and suspended in fresh mDixon liquid medium. Aliquots of cultures were then inoculated in wells of a 96-well plate and adjusted to a starting OD_600_ of 0.1 for each well. The wells were then treated with triacsin C (Cayman Chemical) at various doses and incubated at 32 °C in a shaking incubator. OD values were measured at indicated times using Varioskan Lux (Thermo Scientific).

### 2.5. Isolation of M. pachydermatis Total RNA

The mDixon liquid medium was inoculated with one loop of *M. pachydermatis* and pre-cultured for 5–7 days, then transferred to 100 mL of mDixon medium for a starting OD_600_ of 0.05. The cells were incubated in a shaker at 120 rpm at 32 °C until OD_600_ reached 1–2 and then collected by washing with the mYNB (modified Yeast Nitrogen Base) medium (0.67% yeast nitrogen base (Difco), 2% glycerol, 1% Tween 40, and 25 mM MOPS buffer (pH 6)). To induce gene expression, 10 mL of cell suspension (starting OD_600_ = 1) in mYNB containing 5 mM of each fatty acid (C12:0, C14:0, C16:0, and C18:1) or in mDixon containing 3 μM triacsin C were incubated in L-tubes at 32 °C for 6 h. Before the cells were collected, 0.05% Triton X-100 was added as a surfactant and mixed vigorously to facilitate pellet collection. The supernatants containing each fatty acid were carefully removed, and the pellets for RNA extraction were washed with DEPC water. Total RNA was extracted from each sample using the hot phenol method [32]. The concentration of each isolated RNA was determined using a GeneQuant100 spectrophotometer (GE Healthcare), and RNA quality was confirmed by agarose gel electrophoresis.

### 2.6. cDNA Synthesis and Transcription Level Analysis

Isolated mRNAs were reverse transcribed into cDNAs using ReverTra Ace qPCR Master Mix with gDNA Remover (Toyobo). The mRNA transcription level was analyzed using THUNDERBIRD SYBR qPCR Mix (Toyobo) with StepOne qualitative real-time PCR (qRT-PCR) from Applied Biosystems. The primers used for qRT-PCR are listed in Table S3.

## 3. Results

### 3.1. Identification of Malassezia orthologs of S. cerevisiae FAA1 Genes

**Figure 1:**
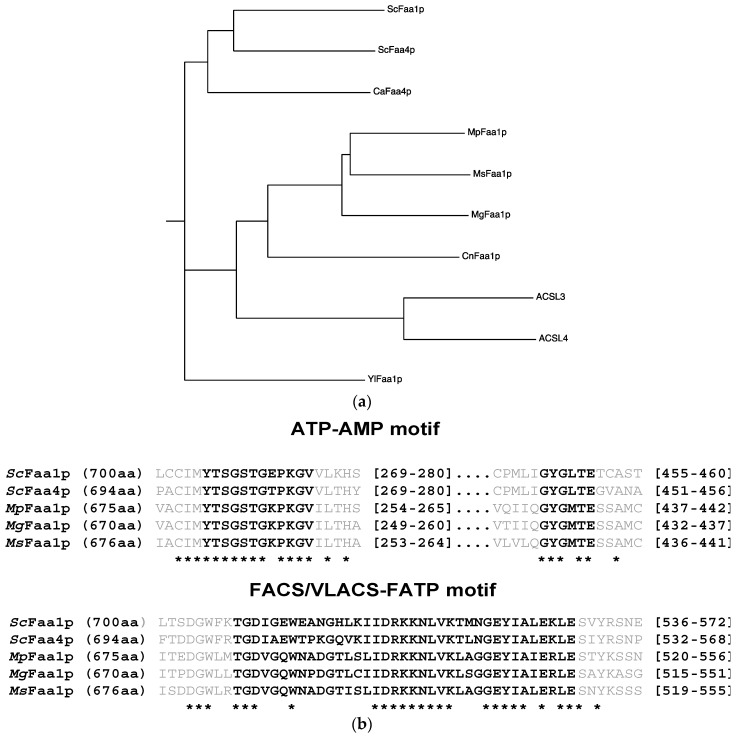
Phylogenetic tree of ACS enzymes in *S. cerevisiae* and *Malassezia* spp. (**a**) Phylogenetic tree of Faa1 proteins in various fungi, constructed using ClustalW and drawn using Phylodendron’s phenogram (http://iubio.bio.indiana.edu/treeapp/treeprint-form.html). *Mp*Faa1p: Malapachy_0054; *Mg*Faa1p: MGL_3626; *Ms*Faa1p: MSYG_3835; *Ca*Faa4p: *Candida albicans* Faa4; *Cn*Faa1p: *Cryptococcus neoformans* Faa1; *Yl*Faa1p: *Yarrowia lipolytica* Faa1; ACSL3: human long-chain fatty acid–CoA ligase 3; ACSL4: human long-chain fatty acid–CoA ligase 4. Scale bar denotes 0.1 substitutions per site. (**b**) Amino acid sequence alignment of Faa1p in *S. cerevisiae* and *M. globosa*, *M. pachydermatis*, and *M. sympodialis*, showing the ATP–AMP motif and FACS/VLACS motif in bold letters. * indicating similar amino acid in all protein aligned in this study. Multiple sequences were aligned using T-Coffee (http://tcoffee.crg.cat/apps/tcoffee/do:regular). Asterisks denote amino acids conserved in all proteins aligned.

To identify orthologs of the *ScFAA1* gene in *Malassezia* spp., *M. pachydermatis*, *M. globose*, and *M. sympodialis* genomic data were searched against the NCBI database using blastp analysis. Faa orthologs of these *Malassezia* species exhibited 36–38% identity with *Sc*Faa1p (Table 1, Figure 1). MGL_3626 of *M. globosa* (*MgFAA1*) and Malapachy_0054 of *M. pachydermatis* (*MpFAA1*) encode 670- and 675-amino acid residue proteins, respectively. For *M. sympodialis* (*MsFAA1*), we identified two highly homologous orthologs: MSY001_1358 (662 amino acids) and MSYG_3835 (676 amino acids). However, the nucleic acid sequences of these orthologs were almost identical. A 14-amino acid sequence spanning residues 438–451 was missing in MSY001_1358, compared with MSYG_3835. We hypothesized that MSY001_1358 and MSYG_3835 are the same gene in the *M. sympodialis* genome. In addition, the amino acid sequences of the three Faa1p orthologs exhibiting the highest identity were found to contain a conserved ATP–AMP motif and FACS/VLACS–FATP motif (Figure 1b). The orthologs from these three species with the second-highest degree of similarity exhibited a lower degree of identity with *Sc*Faa1p (Malapachy_0976: 29%, MGL_0129: 27%, and MSY001_0514: 25%). However, as they exhibited higher identity with *Sc*Faa2p than *Sc*Faa1p, it was concluded that these three genes were not good matches for Faa1p of *Malassezia.*

### 3.2. Malassezia Faa1 Proteins Complement the Function of S. cerevisiae Faa1p and Faa4p

**Figure 2:**
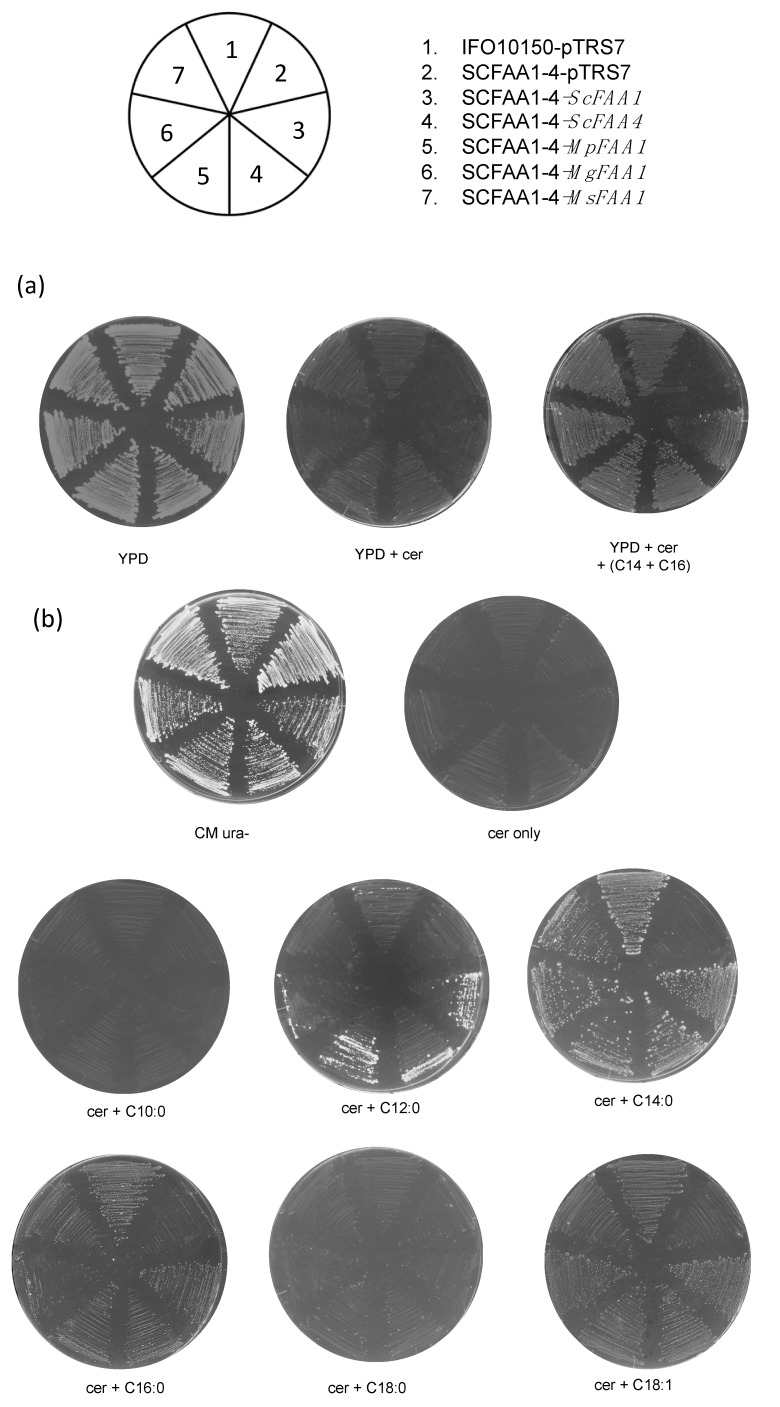

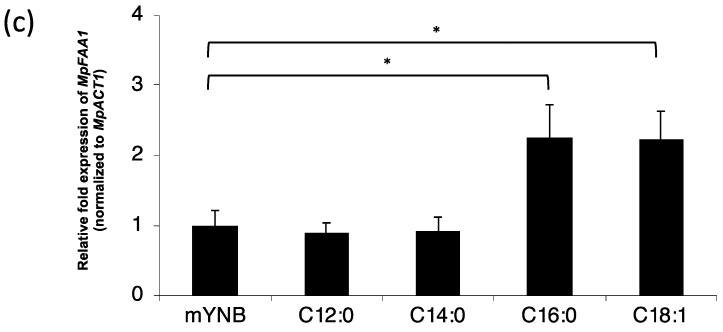
(**a**) Complementation analysis and substrate specificity determination for *MpFAA1*, *MgFAA1*, and *MsFAA1. S. cerevisiae* IFO10150-pTRS7 (*FAA1FAA4)* and its *FAA1* and *FAA4* deletion mutant (SCFAA1-4--pTRS7) as well as SCFAA1-4-*ScFAA1,* SCFAA1-4-*ScFAA4,* SCFAA1-4-*MpFAA1*, SCFAA1-4-*MgFAA1*, and SCFAA1-4-*MsFAA1* were streaked on YPD agar plates containing cerulenin and C14:0 and C16:0 fatty acids. (**b**) Growth on CM ura^−^ agar plates containing C10:0, C12:0, C14:0, C16:0, C18:0, and C18:1 (0.5 mM) and supplemented with 5 μg/mL cerulenin and cultured at 30 °C for 5–10 days. CM ura^−^ agar plates containing cerulenin without fatty acids were used as controls. (**c**) Levels of *MpFAA1* transcripts in mYNB medium containing various fatty acids. *Malassezia pachydermatis* 1879^NT^ was grown in mDixon liquid medium until early/mid-log phase, transferred to mYNB medium containing 5 mM fatty acid (C10:0, C12:0, C14:0, C16:0, C18:0, and C18:1), and incubated for 6 h at 32 °C. RNAs were extracted, reverse transcribed to cDNA, and then analyzed to determine gene expression level by qRT-PCR relative to the expression of *MpACT1* (*n* = 3 experiments, each done in duplicate; * *p* < 0.05).

*ScFAA1* and *ScFAA4* are primary LCFA activators and play important roles in facilitating the utilization of exogenous LCFAs. The deletion of both of these genes caused a growth defect in *S. cerevisiae* in media containing cerulenin and LCFAs [16]. The introduction of *MpFAA1*, *MgFAA1*, and *MsFAA1* into SCFAA1-4 rescued the growth defect in the *S. cerevisiae* mutant in YPD medium containing cerulenin, myristic acid (C14:0), and palmitic acid (C16:0) (Figure 2a). These data indicated that the three *Malassezia FAA1* genes and *ScFAA1* and *ScFAA4* are functionally complementary to loss of *ScFAA1* and *ScFAA4.*

To investigate the substrate specificity of the *Malassezia FAA1* genes, we analyzed the growth of strains SCFAA1-4 harboring *MpFAA1*, *MgFAA1*, and *MsFAA1* in a synthetic medium (CM ura^−^) supplemented with cerulenin and fatty acids with varying numbers of carbons: Capric acid (C10:0), lauric acid (C12:0), C14:0, C16:0, stearic acid (C18:0), and oleic acid (C18:1). On media containing C14:0, C16:0, and C18:1, all the strains except SCFAA1-4-pTRS7 could grow, although the growth on media with C16:0 and C18:1 was slower than that observed in C14:0. SCFAA1-4 strains expressing *MpFAA1*, *MgFAA1, ScFAA1,* and *ScFAA4* showed a slight growth in the C12-supplemented medium, while IFO10150-pTRS7 and SCFAA1-4 expressing *Ms*Faa1p were unable to grow in this medium (Figure 2b).

We also analyzed the transcription of *MpFAA1* in *M. pachydermatis* cultured in mYNB medium with several different fatty acids. Expression of the *MpFAA1* gene in medium containing C16:0 and C18:1 was enhanced approximately 2-fold compared with cells grown in mYNB medium only. These data indicated that *MpFAA1* expression was induced by addition of at least C16:0 and C18:1 (Figure 2c).

### 3.3. Inhibition of Malassezia Faa1p by Triacsin C

**Figure 3:**
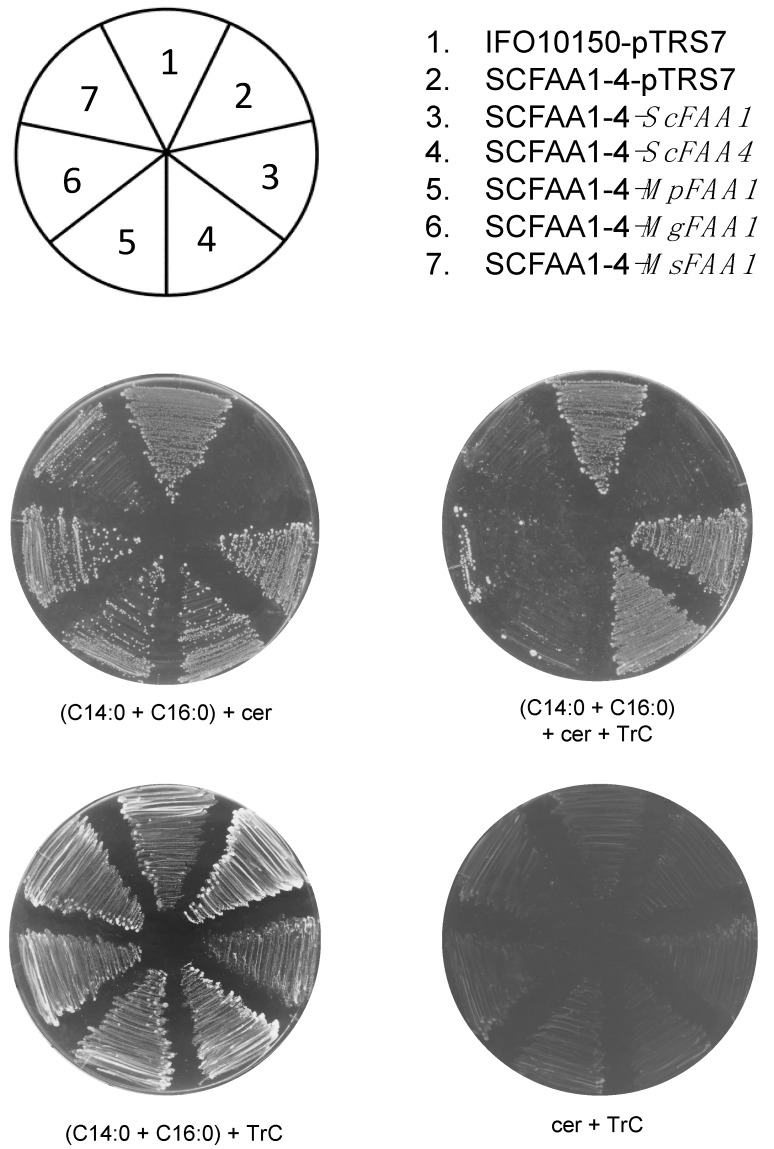
Triacsin C blocks the rescue of *Malassezia FAA1* in *S. cerevisiae faa1* and *faa4* mutant. A CM ura^−^ plate containing cerulenin (5 μg/mL), C14:0 + C16:0 (each 0.5 mM), and triacsin C (3 μM) was prepared. An agar plate containing C14:0 + C16:0 and triacsin C without cerulenin, and an agar plate containing cerulenin and triacsin C were used as positive and negative controls, respectively. *S. cerevisiae* strains were grown at 30 °C for 3–5 days.

Triacsin C is known as an inhibitor of bacterial and mammal ACSs [23,24]. To investigate the effect of triacsin C on *Malassezia* Faa1p, SCFAA1-4 strains expressing *MpFAA1*, *MgFAA1*, and *MsFAA1* were cultivated in a medium containing cerulenin, LCFAs, and triacsin C. Triacsin C blocked the rescue of *Malassezia* Faa1 proteins in the SCFAA1-4 mutants. In contrast, the growth of *S. cerevisiae* IFO10150 and SCFAA1-4 strains expressing *ScFAA1* and *ScFAA4* was not affected by triacsin C (Figure 3). These data suggested that triacsin C inhibits the activity of *Malassezia* Faa1 proteins, not *Sc*Faa1 and *Sc*Faa4 proteins.

**Figure 4:**
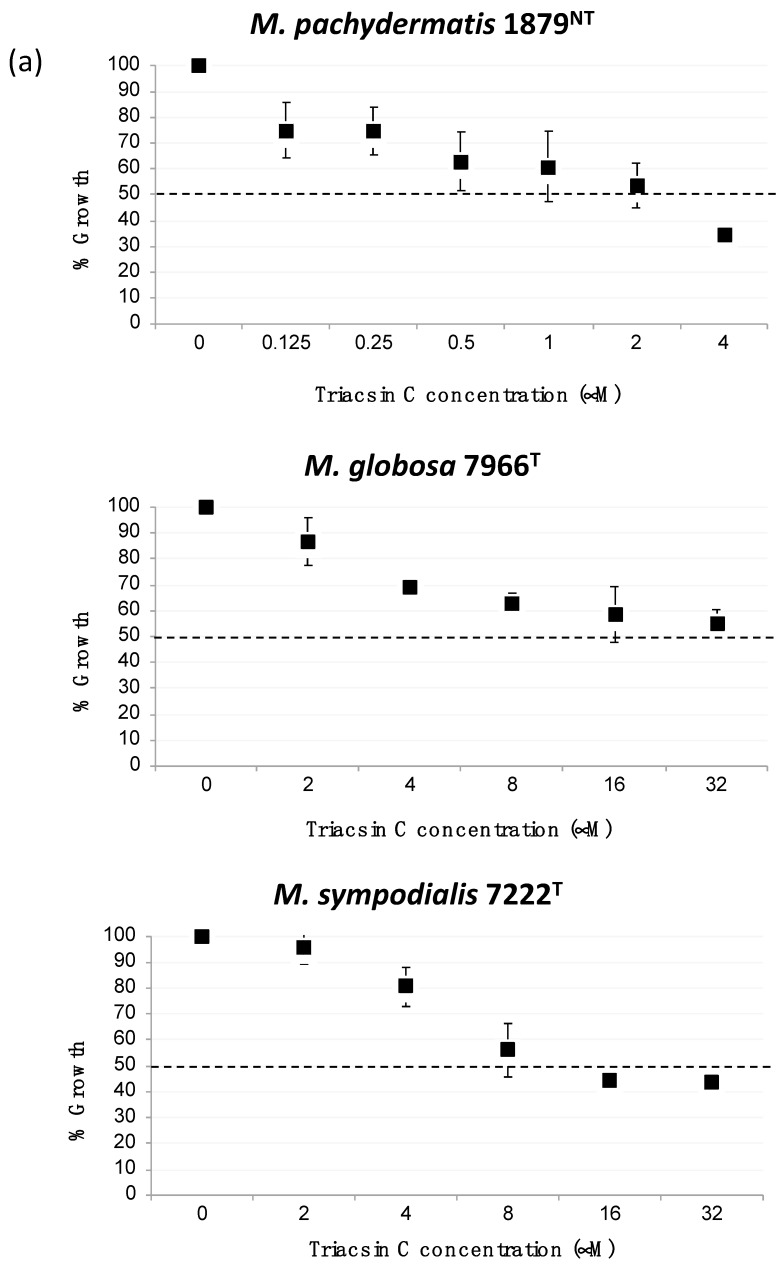

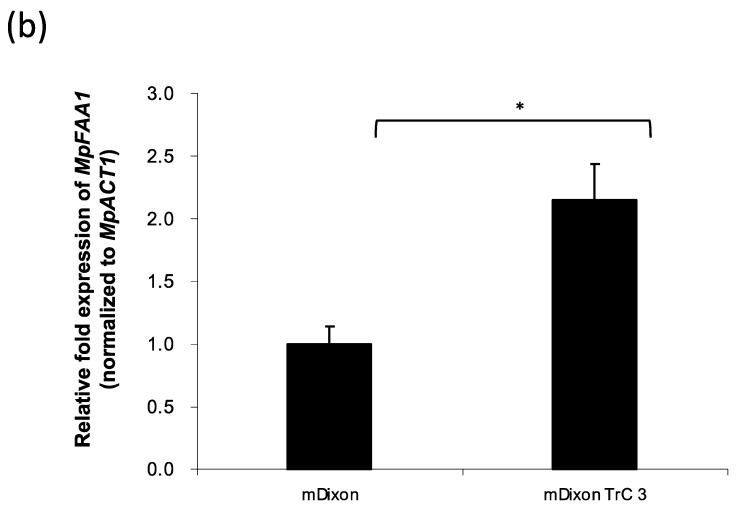
(**a**) Inhibition of the growth of *M. pachydermatis* 1879^NT^, *M. globosa* 7966^T^, and *M. sympodialis* 7222^T^ by triacsin C. All the three *Malassezia* strains were grown at 32 °C in mDixon liquid medium supplemented with triacsin C in a 96-well plate with a starting OD_600_ of 0.1. After 48 h, the OD was measured at 600 nm using a microplate reader (*n* > 3 experiments, each done in triplicate). (**b**) *M. pachydermatis* 1879^NT^ was grown in mDixon liquid medium until early/mid-log phase, transferred to a fresh liquid mDixon medium containing 3 μM triacsin C, and incubated for 6 h at 32 °C. RNAs were extracted, reverse transcribed to cDNAs, and then assayed to determine gene expression levels relative to the expression of *MpACT1* using qRT-PCR analysis (*n* = 3 independent experiments, each done in duplicate; * *p* < 0.05).

To assess the inhibitory effect of triacsin C on the growth of *Malassezia* spp., we cultured the three species of this yeast in mDixon medium with LCFAs and triacsin C. The growth of *M. pachydermatis*, *M. globosa*, and *M. sympodialis* decreased approximately 50% in the presence of triacsin C at 2 μM, 32 μM, and 8 μM (MIC_50_), respectively (Figure 4a). We hypothesized that the suppressive effect of triacsin C on the growth of the *Malassezia* species was due to interference with fatty acid activation. We also analyzed transcription of *MpFAA1* in the presence of triacsin C and found that the expression of *MpFAA1* was induced by the inhibitor (Figure 4b). These data suggested that triacsin C-mediated blockade of fatty acid activation induced an increase in the production of Faa1p, thus upregulating the expression of *MpFAA1.*

## 4. Discussion

Fatty acid uptake and activation are thought to be essential processes in *Malassezia* spp. The absence of genes encoding FASs results in the absolute requirement that *Malassezia* cells obtain LCFAs from the skin of the hosts in order to grow. Internalized LCFAs are activated to long-chain fatty acyl–CoA by ACSs by various metabolic pathways, including membrane lipid synthesis and other metabolic pathways that could be crucial for pathogenicity.

The details of fatty acid uptake and activation in *Malassezia* spp. remain unclear. In the present study, we identified *FAA1* genes encoding ACSs in three *Malassezia* species (Figure 1a), and we introduced these genes into an *S. cerevisiae faa1-faa4* double mutant SCFAA1-4. The results of these experiments suggested that *MpFAA1*, *MgFAA1*, and *MsFAA1* function similar to *ScFAA1* and *ScFAA4* in the activation of exogenous LCFAs (Figure 2). In addition, although other *Malassezia* orthologs (Malapachy_0976, MGL_0129, and MSY001_0514) that exhibited the second-highest similarity to *Sc*Faa1p were introduced into SCFAA1-4, these genes could not restore the growth of this mutant in media containing cerulenin and fatty acids (data not shown). The disruption of *FAA4* in the pathogenic fungus *Candida albicans* is lethal in a medium containing 14:0 and 16:0 fatty acids in the presence of cerulenin [33]. Similar to *C. albicans*, the *Malassezia* spp. genome may contain only one gene encoding an exogenous LCFA-activating enzyme.

Unexpectedly, *S. cerevisiae* strains SCFAA1-4 expressing *MpFAA1* and *MgFAA1* were able to grow slightly in medium containing cerulenin and C12:0 fatty acid, although the IFO10150--pTRS7 (*FAA1FAA4)* strain could not (Figure 2b). SCFAA1-4 strains expressing *ScFAA1* and *ScFAA4* also showed the ability to grow a little in C12-supplemented medium. Thus, the overexpression of these genes may lead to the activation of C12:0 for the minimal growth although the C12:0-substrate affinity of these LACSs are quite low. Nevertheless, since SCFAA1-4 strain expressing *MsFAA1* is unable to grow in C12:0, this result assumed that *Ms*Faa1 protein could not recognize C12:0 fatty acid as a substrate.

A comparison of the amino acid sequences of *Mp*Faa1, *Mg*Faa1, and *Ms*Faa1 proteins revealed no remarkable differences. In this experiment, we did not perform codon optimization of *Malassezia FAA1* genes for expression in *S. cerevisiae.* This could be one reason why these genes could not fully restore the *FAA1* and *FAA4* mutations in SCFAA1-4. To explain this discrepancy, further experiments investigating whether the expression level of *Malassezia* Faa1 proteins is sufficient to activate LCFAs in *S. cerevisiae* will be necessary.

As triacsin C is known to affect mammalian cells, this compound was used to block the function of *Malassezia* Faa1 proteins in *S. cerevisiae*. We hypothesized that Faa1p and Faa4p are potential drug targets for therapies against *Malassezia* spp., which are incapable of synthesizing fatty acids endogenously. Triacsin C prevented the rescue of SCFAA1-4 expression of *MpFAA1*, *MgFAA1*, and *MsFAA1* in a medium containing cerulenin and exogenous LCFAs. As expected, triacsin C did not suppress the growth of *S. cerevisiae* IFO10150 as well as SCFAA1-4 strains harboring *ScFAA1* and *ScFAA4*, even when de novo fatty acid synthesis pathways were inactive. A previous study reported that 10 μM of triacsin C did not affect the growth of *S. cerevisiae* strains harboring the *FAA1* and *FAA4* genes in a synthetic medium containing cerulenin and C14:0 fatty acid [28]. Thus, our results provide evidence that *Malassezia* Faa1 proteins are targets of triacsin C, but *S. cerevisiae* Faa1p and Faa4p are not.

To confirm that triacsin C inhibits the growth of *Malassezia* spp., the drug was added to cultures of three *Malassezia* species. Triacsin C reduced the growth of all three species in a dose-dependent manner in the mDixon medium. However, triacsin C was not totally lethal to the *Malassezia* species examined. This suggests that *Malassezia* species have an alternative mechanism for LCFA activation involving triacsin C-resistant ACSs. It is also possible that triacsin C does not completely inhibit the activity of *Malassezia* Faa1 proteins due to low binding affinity. To address this possibility, it will be necessary to conduct in vitro enzyme assays using cell-free lysate and purified *Malassezia* Faa1 proteins. Nonetheless, in this work, we set the scope to *Malassezia FAA1* gene complementation in *S. cerevisiae* and *Malassezia* growth reduction by triacsin C. Further experiments will determine the target proteins of triacsin C in *Malassezia* spp.

ACSs are conserved in all organisms, including humans. Triacsin C is often used in studies of lipid metabolism in various human diseases. A previous report showed that triacsin C inhibits the incorporation of oleic acid into lipids and glycerolipids in human fibroblasts [24]. Triacsin C is also a potent inhibitor of microsomal and mitochondrial long-chain ACSs in human hepatocytes [27]. However, to date, there are no other reports describing triacsin C toxicity in humans (particularly skin toxicity) or therapeutic applications for human or animal skin. Research in mice demonstrated that at low doses, triacsin C can be used to control the growth of cancer cells, with no negative side effects [34]. We demonstrated that triacsin C partially inhibits the activity of Faa1 proteins in *Malassezia* spp. Therefore, changes to the structure of this compound will be required to develop a more effective anti-*Malassezia* drug for treating skin diseases. In conclusion, our data demonstrate that *Malassezia* Faa1 proteins are important targets in efforts to develop novel anti-*Malassezia* drugs. Our results indicate that triacsin C and its derivatives are potentially ideal candidates.

## Figures and Tables

**Table 1:** Acyl-CoA synthetase orthologs identified in the *Malassezia* spp. genome using the National Center for Biotechnology Information (NCBI) conserved domain database.

Gene Name	Accession No.	*Malassezia* Gene ID	Protein Size	Description	% Identity to		Query Cover to	
					*ScFAA1*	*ScFAA4*	*ScFAA1*	*ScFAA4*
*MpFAA1*	XP_017991190.1	Malapachy_0054	675 aa	long-chain fatty acid ligase	38	37	87	99
*MgFAA1*	XP_001729159.1	MGL_3626	670 aa	hypothetical protein	37	36	97	98
*MsFAA1*	SHO79486.1	MSYG_3835	676 aa	similar to *S. cerevisiae FAA4*	37	39	99	98
*MsFAA1*	XP_018739945.1	MSY001_1358	662 aa	hypothetical protein	36	38	99	98

## Supplementary Materials

The following are available online at https://www.mdpi.com/2309-608X/5/4/88/s1, Table S1: *Saccharomyces cerevisiae* strains used in this study, Table S2: Plasmids used in this study, Table S3: Primers used in this study.
