# Supplementary material for: Long-Chain Acyl-CoA Synthetase is Associated with the Growth of Malassezia spp
Source: J Fungi (Basel). 2019 Sep 21;5(4):88. doi: 10.3390/jof5040088 (PMC6958399; doi:10.3390/jof5040088)
Supplement: Supplementary file 1 [file jof-05-00088-s001.pdf]

Table S1
Saccharomyces cerevisiae strains used in this study.

Strain	Genotype	Source of reference
IFO10150	*MATa ste-VC9 ura3-52 trp1-289 his3-Δ1 leu2-3112 pTRS7*	(Tejima, et al. 2017)
SCFAA1-4	*SCFAA1-4-Ura MATa ste-VC9 ura3-52 trp1-289 his3-Δ1 leu2-3112 Scfaa1Δ::dpl200 Scfaa4Δ::dpl200*	(Tejima, et al. 2017)
SCFAA1-4-pTRS7	*SCFAA1-4 MATa ste-VC9 ura3-52 trp1-289 his3-Δ1 leu2-3112 Scfaa1Δ::dpl200 Scfaa4Δ::dpl200 pTRS7*	(Tejima, et al. 2017)
SCFAA1-4-ScFAA1	*SCFAA1-4 MATa ste-VC9 ura3-52 trp1-289 his3-Δ1 leu2-3112 Scfaa1Δ::dpl200 Scfaa4Δ::dpl200 pTRS7-ScFAA1*	This study
SCFAA1-4-ScFAA4	*SCFAA1-4 MATa ste-VC9 ura3-52 trp1-289 his3-Δ1 leu2-3112 Scfaa1Δ::dpl200 Scfaa4Δ::dpl200 pTRS7-ScFAA4*	This study
SCFAA1-4-MgFAA1	*SCFAA1-4 MATa ste-VC9 ura3-52 trp1-289 his3-Δ1 leu2-3112 Scfaa1Δ::dpl200 Scfaa4Δ::dpl200 pTRS7-MgFAA1*	This study
SCFAA1-4-MpFAA1	*SCFAA1-4 MATa ste-VC9 ura3-52 trp1-289 his3-Δ1 leu2-3112 Scfaa1Δ::dpl200 Scfaa4Δ::dpl200 pTRS7-MgFAA1*	This study
SCFAA1-4-MsFAA1	*SCFAA1-4 MATa ste-VC9 ura3-52 trp1-289 his3-Δ1 leu2-3112 Scfaa1Δ::dpl200 Scfaa4Δ::dpl200 pTRS7-MgFAA1*	This study

*IFO: Institution for Fermentation, Osaka, Japan

Table S2

Plasmids used in this study.

Plasmid	Description	Source of reference
pTRS7	*S. cerevisiae* cloning vector	(Tejima, 2017)
pTRS7-ScFAA1	A plasmid for expression of *S. cerevisiae FAA1* into SCFAA1-4	This study
pTRS7-ScFAA4	A plasmid for expression of *S. cerevisiae FAA4* into SCFAA1-4	This study
pTRS7-MpFAA1	A plasmid for expression of *M. pachydermatis FAA1* into SCFAA1-4	This study
pTRS7-MgFAA1	A plasmid for expression of *M. globosa FAA1* into SCFAA1-4	This study
pTRS7-MsFAA1	A plasmid for expression of *M. sympodialis FAA1* into SCFAA1-4	This study

Table S3

Primers used in this study.

Restriction endonuclease sites are underlined.

Name	Primer sequence (5′-3′)
ScFAA1-F	TTGTCGACGTAAGTATAGAAAGTTCTTGTTGT
ScFAA1-R	TTGCGGCCGCGAAAAAGTGCTTTAGTATGATGAGGC
ScFAA4-F	TTGGATCCCCCTCTGAGTTGACATAAAAGCG
ScFAA4-R	TTGCGGCCGCGAGAAGAAACTATGTATTGTGCAAGT
MpFAA1-F	TTGTCGACGAGAACGTAAGCAGGCAA
MpFAA1-R	TTCCCGGGCCGGACGAACCACGCTAT
MgFAA1-F	TTGAATTCTCTAATCGGCCTTCCGTT
MgFAA1-R	TTGGATCCACGCACGGATTGGCTATG
MsFAA1-F	TTCCCGGGAGCAGTCACGTTTTCAA
MsFAA1-R	TTACTAGTGGCCACGCCACACCGTAAT
RT-PCR(MpFAA1-F)	AAGAAGGTAGGTGGCGAAGAGA
RT-PCR(MpFAA1-R)	GCTCAAACCACTCGCAAAGA
RT-PCR(MpACT1-F)	CTTACGAACCAACCTGTGGTGA
RT-PCR(MpACT1-R)	ACATTTCGGCGTGTCTTGG
